# Supplementary material for: Dissecting the dynamic transcriptional landscape of early T helper cell differentiation into Th1, Th2, and Th1/2 hybrid cells
Source: Front Immunol. 2022 Aug 16;13:928018. doi: 10.3389/fimmu.2022.928018 (PMC9424495; doi:10.3389/fimmu.2022.928018)
Supplement: Supplementary file 1 [file Image_1.pdf]

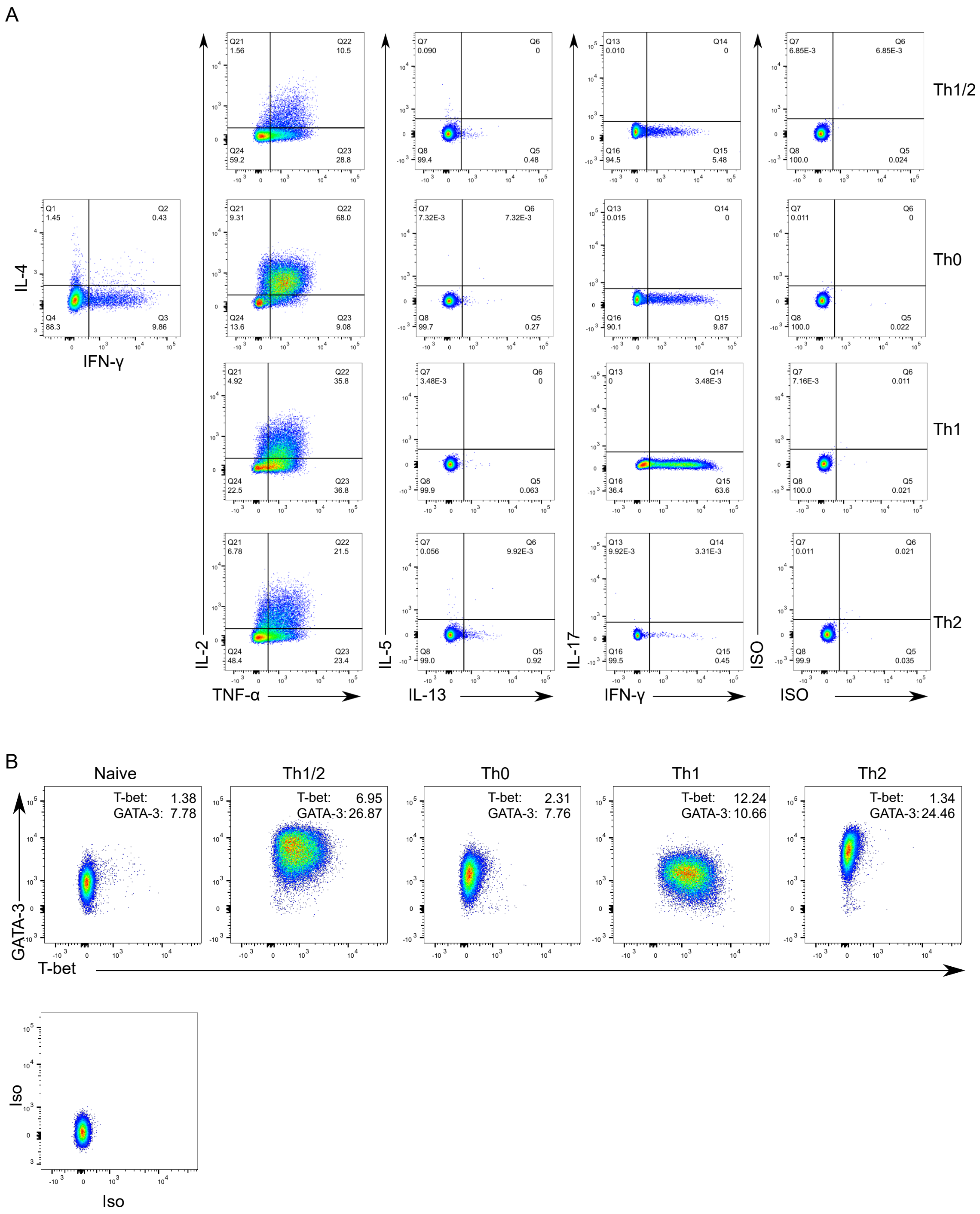

Figure S1: Flow-cytometric evaluation of Th cell subsets. (A) Flow cytometry analysis of signature cytokines of mouse-derived naïve CD4+ Th cells exposed to polarizing Th1, Th0, Th2 and Th1/2 conditions 120 hours after activation. (B) Staining for signature transcription factors of Th cell subsets for the same conditions as in (A). Geometric mean indices for T-bet and GATA-3 are shown.
