# Supplementary material for: Dissecting the dynamic transcriptional landscape of early T helper cell differentiation into Th1, Th2, and Th1/2 hybrid cells
Source: Front Immunol. 2022 Aug 16;13:928018. doi: 10.3389/fimmu.2022.928018 (PMC9424495; doi:10.3389/fimmu.2022.928018)
Supplement: Supplementary file 2 [file Image_2.pdf]

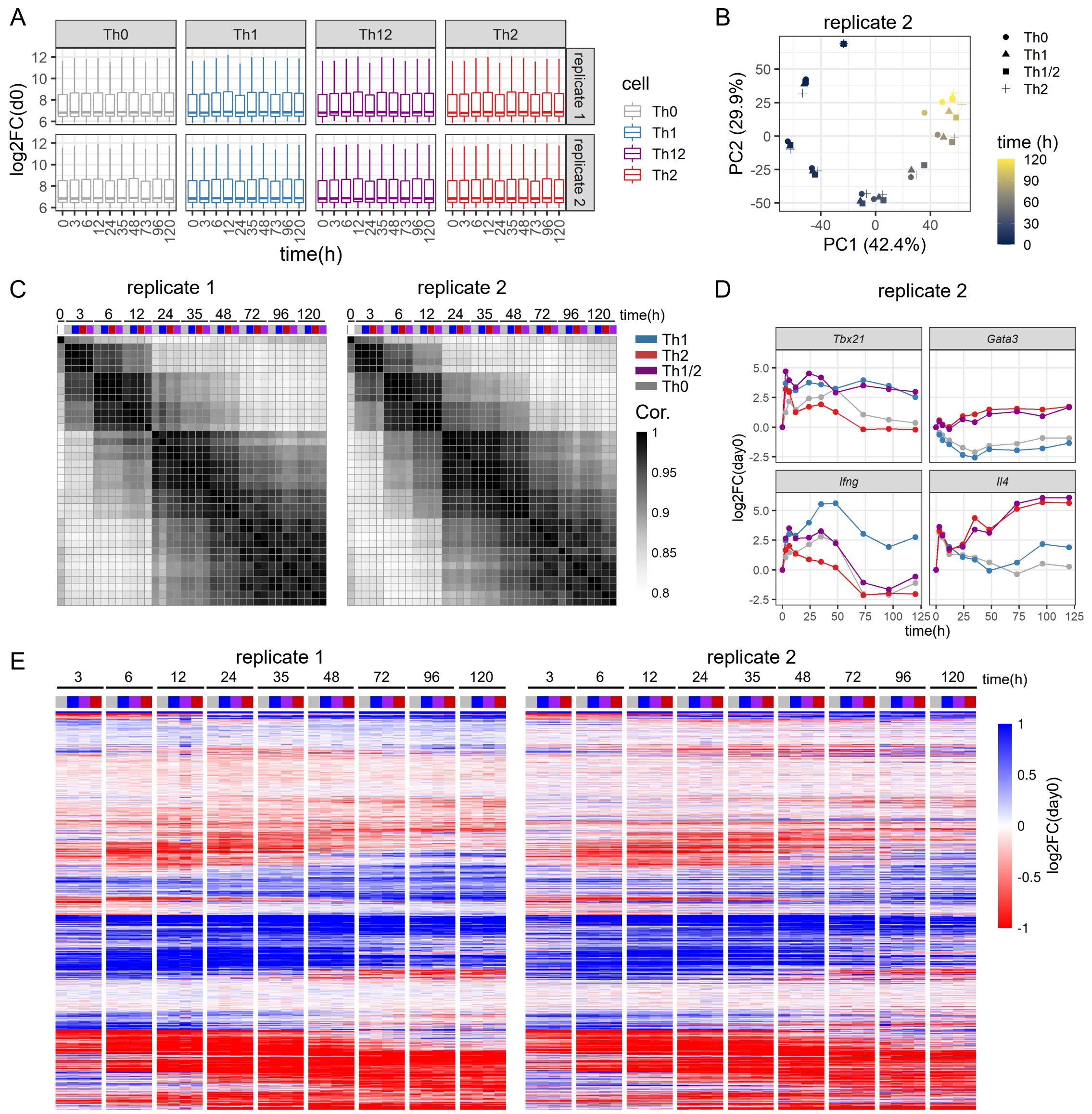

Figure S2: Exploratory data analysis and quality controls. (A) Gene expression distributions for individual samples after data cleaning and normalization. (B) PCA for replicate 2. (C) Correlation heatmaps (Pearson correlation) for all time points and conditions. (D) Transcription factor and signature cytokine dynamics for Th cell subsets in replicate 2. (E) Heatmap of all expressed genes (replicate 1 and 2). Genes in both replicates are sorted using hierarchical clustering based on output from replicate 1. Cell types are indicated by color as in panel (A). Expression values are normalized to day 0.
