# Supplementary material for: Dissecting the dynamic transcriptional landscape of early T helper cell differentiation into Th1, Th2, and Th1/2 hybrid cells
Source: Front Immunol. 2022 Aug 16;13:928018. doi: 10.3389/fimmu.2022.928018 (PMC9424495; doi:10.3389/fimmu.2022.928018)
Supplement: Supplementary file 3 [file Image_3.pdf]

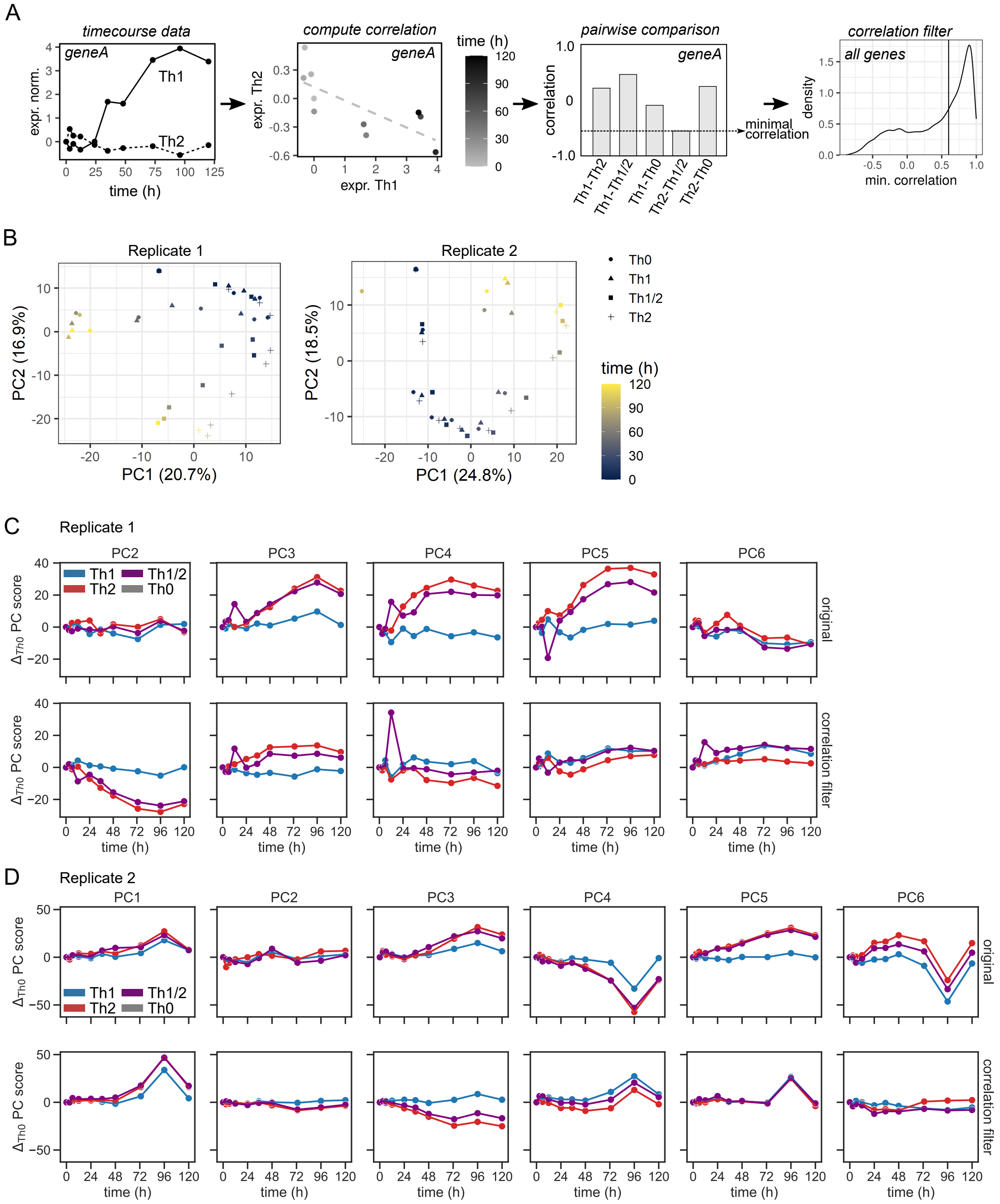

Figure S3: Workflow to remove highly correlated genes. (A) Gene-wise correlation was computed for all pairwise comparisons between samples. All genes with correlation coefficient exceeding a threshold value in at least one comparison were removed (cf. Methods). (B) Principal component (PC) analysis of the differentiation time course after removal of highly correlated genes. Cell subsets are indicated by marker shape. Time of measurement is indicated by color. (C-D) Time-evolution of principal components with and without removal of highly correlated genes. Shown are PC values subtracted by the value of the Th0 condition for replicate 1 (C) and replicate 2 (D).
