# Supplementary material for: Dissecting the dynamic transcriptional landscape of early T helper cell differentiation into Th1, Th2, and Th1/2 hybrid cells
Source: Front Immunol. 2022 Aug 16;13:928018. doi: 10.3389/fimmu.2022.928018 (PMC9424495; doi:10.3389/fimmu.2022.928018)
Supplement: Supplementary file 4 [file Image_4.pdf]

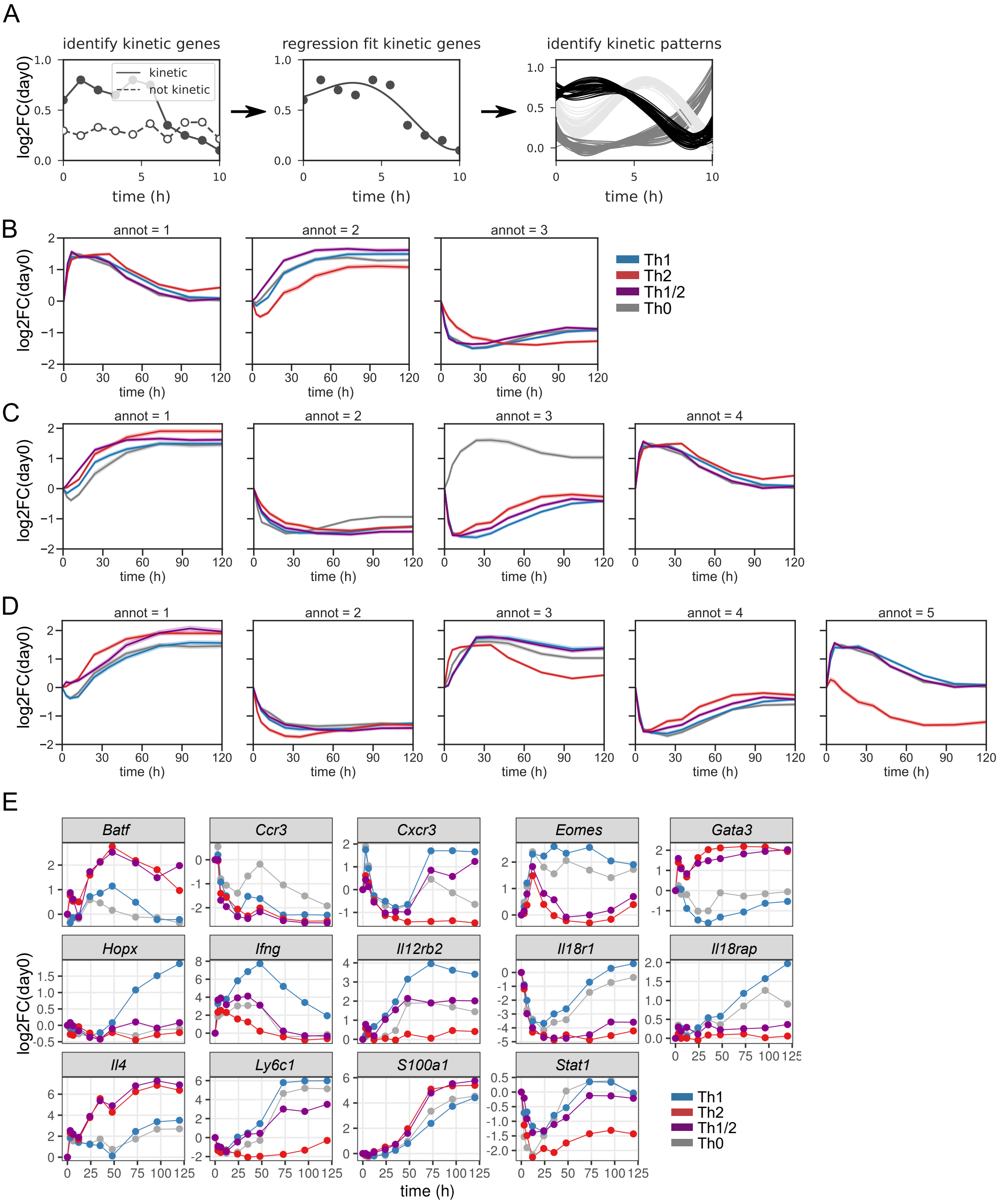

Figure S4: Supplementary analysis of kinetic gene expression profiles. (A) Regression-based MaSigPro workflow to analyze kinetic gene expression profiles . Kinetic genes are identified by regression and then clustered based on gene-gene correlation (cf. Methods). (B-D) Kinetic gene expression patterns computed setting the number of clusters to n=3,4 and 5. Shown are average gene expression values in each kinetic cluster for all cell types as indicated by color. (E) Expression kinetics of genes that exhibit cluster switches. Shown are genes with different kinetic cluster assignments between at least two cell types, and which are differentially expressed in at least one comparison (cf. Table S1).
