# Supplementary material for: Dissecting the dynamic transcriptional landscape of early T helper cell differentiation into Th1, Th2, and Th1/2 hybrid cells
Source: Front Immunol. 2022 Aug 16;13:928018. doi: 10.3389/fimmu.2022.928018 (PMC9424495; doi:10.3389/fimmu.2022.928018)
Supplement: Supplementary file 5 [file Image_5.pdf]

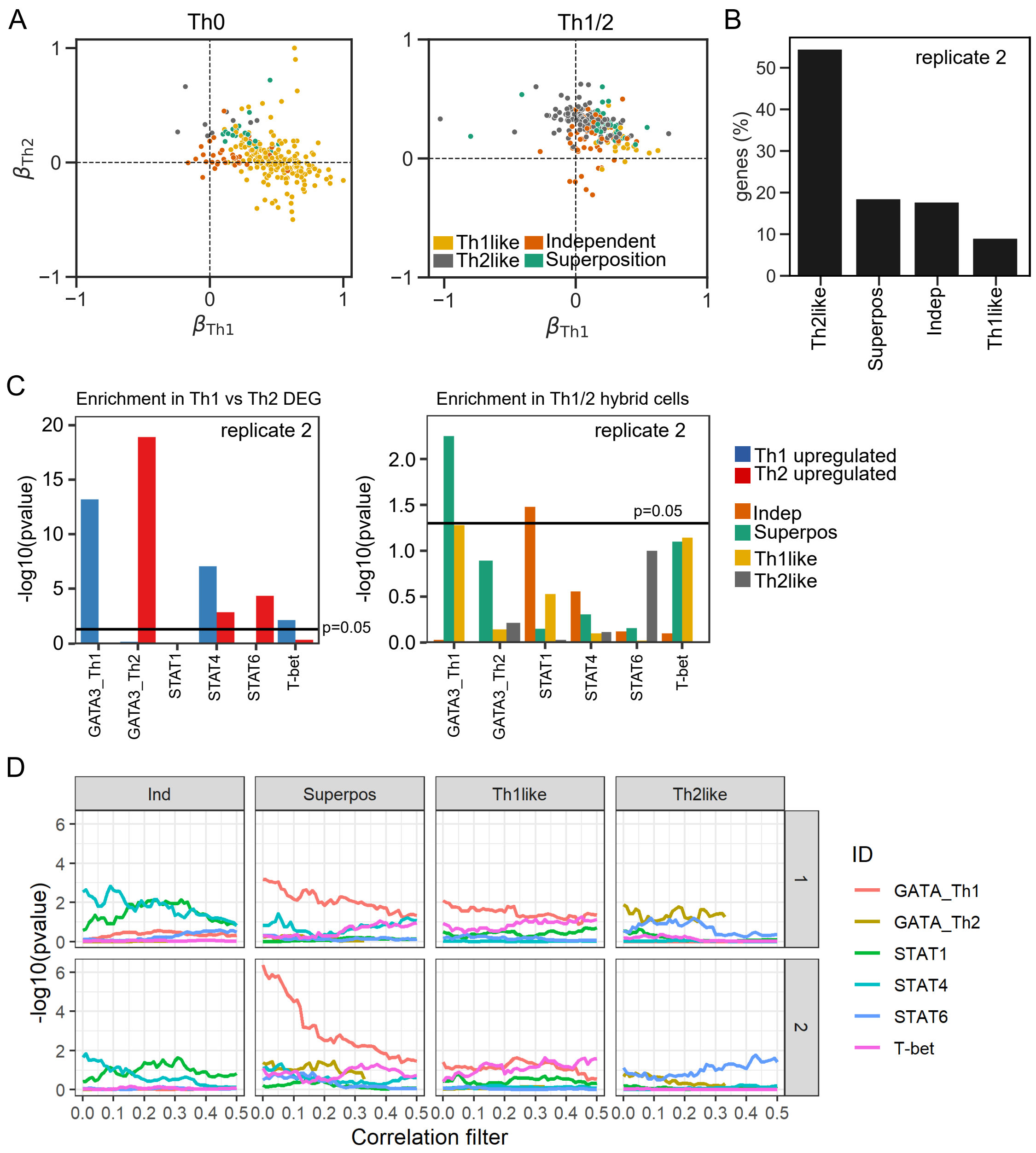

Figure S5: Supplementary analysis of gene expression profiles in Th1/2 hybrid cells. (A) Regression coefficients β_1 and β_2 of the linear regression model employed to identify superimposed, Th1like, Th2like and independent genes in Th0 and Th1/2 cells (cf. Methods and Figure 4A). Coefficients were normalized to the maximum value. (B) Category assignment into independent, superimposed, Th1like and Th2like genes for replicate 2 (cf. Figure 4C). (C) Enrichment results of transcription-factor target gene-sets for replicate 2 (cf. Figure 4D). (D) Enrichment of transcription-factor target gene-sets for different values of the correlation filter in replicate 1 and replicate 2 (cf. Methods).
