# Supplementary material for: Dissecting the dynamic transcriptional landscape of early T helper cell differentiation into Th1, Th2, and Th1/2 hybrid cells
Source: Front Immunol. 2022 Aug 16;13:928018. doi: 10.3389/fimmu.2022.928018 (PMC9424495; doi:10.3389/fimmu.2022.928018)
Supplement: Supplementary file 6 [file Image_6.pdf]

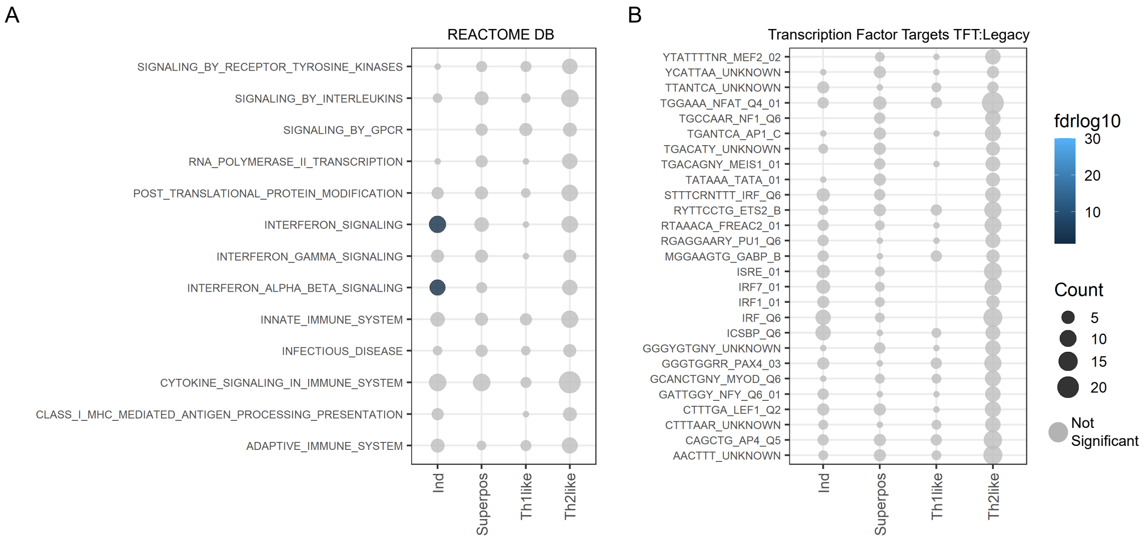

Figure S6: Pathway enrichment analysis for pairwise comparisons of independent, superimposed, Th1like and Th2like genes as derived from the linear model analysis (cf. Figure 4, main text). Pathways were analysed for REACTOME and Transcription Factor Target data bases from msigdbr.
